# Risk factors for pain after total hip arthroplasty: a systematic review

**DOI:** 10.1186/s42836-023-00172-9

**Published:** 2023-04-03

**Authors:** Bo Zhang, Sandesh Rao, Kevin L. Mekkawy, Rafa Rahman, Anzar Sarfraz, Lauren Hollifield, Nick Runge, Julius K. Oni

**Affiliations:** Department of Orthopaedic Surgery, The Johns Hopkins University, 601 North Caroline Street, Baltimore, MD 21287 USA

**Keywords:** Total hip arthroplasty, Systematic review, Pain, Risk factors

## Abstract

**Background:**

Approximately 23% of patients develop hip pain after total hip arthroplasty (THA). In this systematic review, we aimed to identify risk factors associated with postoperative pain after THA to optimize preoperative surgical planning.

**Methods:**

Six literature databases were searched for articles published from January 1995 to August 2020. Controlled trials and observational studies that reported measurements of postoperative pain with assessments of preoperative modifiable and non-modifiable risk factors were included. Three researchers performed a literature review independently.

**Results:**

Fifty-four studies were included in the study for analysis. The most consistent association between worse pain outcomes and the female sex is poor preoperative pain or function, and more severe medical or psychiatric comorbidities. The correlation was less strong between worse pain outcomes and preoperative high body mass index value, low radiographic grade arthritis, and low socioeconomic status. A weak correlation was found between age and worse pain outcomes.

**Conclusions:**

Preoperative risk factors that were consistently predictive of greater/server postoperative pain after THA were identified, despite the varying quality of studies that prohibited the arrival of concrete conclusions. Modifiable factors should be optimized preoperatively, whereas non-modifiable factors may be valuable to patient education, shared decision-making, and individualized pain management.

## Introduction

Total hip arthroplasty (THA) is highly effective in alleviating pain, restoring function, and improving quality of life in patients with severe hip arthritis. However, approximately 23% of patients develop hip pain after THA [1–4]. Some may even experience no improvement or worse pain postoperatively [2]. Known causes of postoperative pain include loosening of implants, infection, periprosthetic fracture, and soft-tissue abnormalities [3]. However, many cases cannot be explained by radiographic or mechanical abnormalities.

Hip pain following THA is often exacerbated by using stairs, walking on uneven surfaces, sitting for extended periods of time, and standing from a seated position. Factors that may influence hip pain involve both modifiable and non-modifiable risk factors. Modifiable risk factors include body mass index (BMI), certain medical and psychological comorbidities, and select socioeconomic variables. Non-modifiable risk factors may cover age, sex, and race. Identification of these modifiable risk factors of increased postoperative pain is critical in guiding preoperative optimization. Understanding non-modifiable risk factors also has added value in determining surgical expectations, individualizing pain management, and guiding the informed decision-making process.

This systematic review aimed to determine preoperative risk factors that are associated with post-THA pain. Hernández et al. reviewed studies prior to 2013 for predictive factors in total knee and total hip replacements [5]. However, THA and TKA are fundamentally distinct surgeries with well-documented differences in outcomes [6]. To our knowledge, there has been no systematic evaluation of risk factors for postoperative pain specific to THA.

## Methods

### Search strategy

We developed a comprehensive search strategy according to the Preferred Reporting Items for Systematic Reviews and Meta-Analyses guidelines with the assistance of a trained librarian. A systematic search was conducted in the PubMed (Medline), Scopus, Web of Science, Cumulative Index of Nursing and Allied Health Literature Plus (EBSCO), Embase, and Cochrane databases by using title, abstract, keywords, and medical subject headings (MeSH). MeSH terms included arthroplasty, replacement, total hip, THA, follow-up, risk assessment, risk factors, reinforcing factors, predictors, pain, postoperative, chronic, long-term, and pain measurement. Title, abstract, and keyword search terms were "hip arthroplasty, THA, THR, hip replacement, OR total hip" AND "after, continue, post, recur, ongoing, chronic, persistent, OR long term" AND "pain" AND "risk, predict, factor, associated, correlate, effect, affect, OR influence". The search strategy was tailored to and optimized for each database.

### Inclusion and exclusion criteria

Our review screened for prospective controlled trials, prospective and retrospective observational cohort studies, and case–control studies. The inclusion criteria were full-text articles published between January 1995 and August 2020 with postoperative pain outcomes in only adult THA cases for osteoarthritis with respect to preoperative risk factors. Studies of patients undergoing THA in combination with other large-joint replacement surgeries were included if data specific to THA were provided. The literature review was performed independently by three researchers. Search results were reviewed in order of title, abstract, then full text, and excluded if they did not meet all criteria. Additional relevant studies were identified through a manual search of the bibliographies of selected studies. All other studies that did not assess risk factors, pain, risk factors for pain, or specific THA data were excluded from this analysis.

### Bias assessment

Criteria for bias are not well described for systematic reviews of observational studies. We performed a bias assessment for each article based on study design and characteristics. The bias assessment was based on (1) loss to follow-up of <20% before 1 year or <30% after 1 year; (2) consecutive patient selection; (3) multicenter recruitment; and (4) use of univariable or multivariable analysis to adjust for confounders. Studies were rated as high risk (meeting zero or one criterion), medium risk (meeting two criteria), or low risk (meeting three or more criteria). If it was unclear whether a study met a criterion, we assumed that it did not.

### Data

Data extracted included study design, patient characteristics, preoperative risk factors, study duration, outcome measurements, postoperative pain outcomes, and criteria for bias assessment. Results for each factor were compared across the studies. Medical or psychological comorbidities, BMI, preoperative pain, and certain socioeconomic elements were considered modifiable factors; whereas, age, sex, and race were deemed as non-modifiable. A descriptive analysis was performed as the heterogeneity of outcome measures and study characteristics prohibited a meaningful meta-analysis.

### Patient and public involvement

No patients were involved in determining the research question, outcome measures, or study design. There are no plans to involve patients in the dissemination of research findings.

## Results

### Study characteristics

**Fig. 1:**
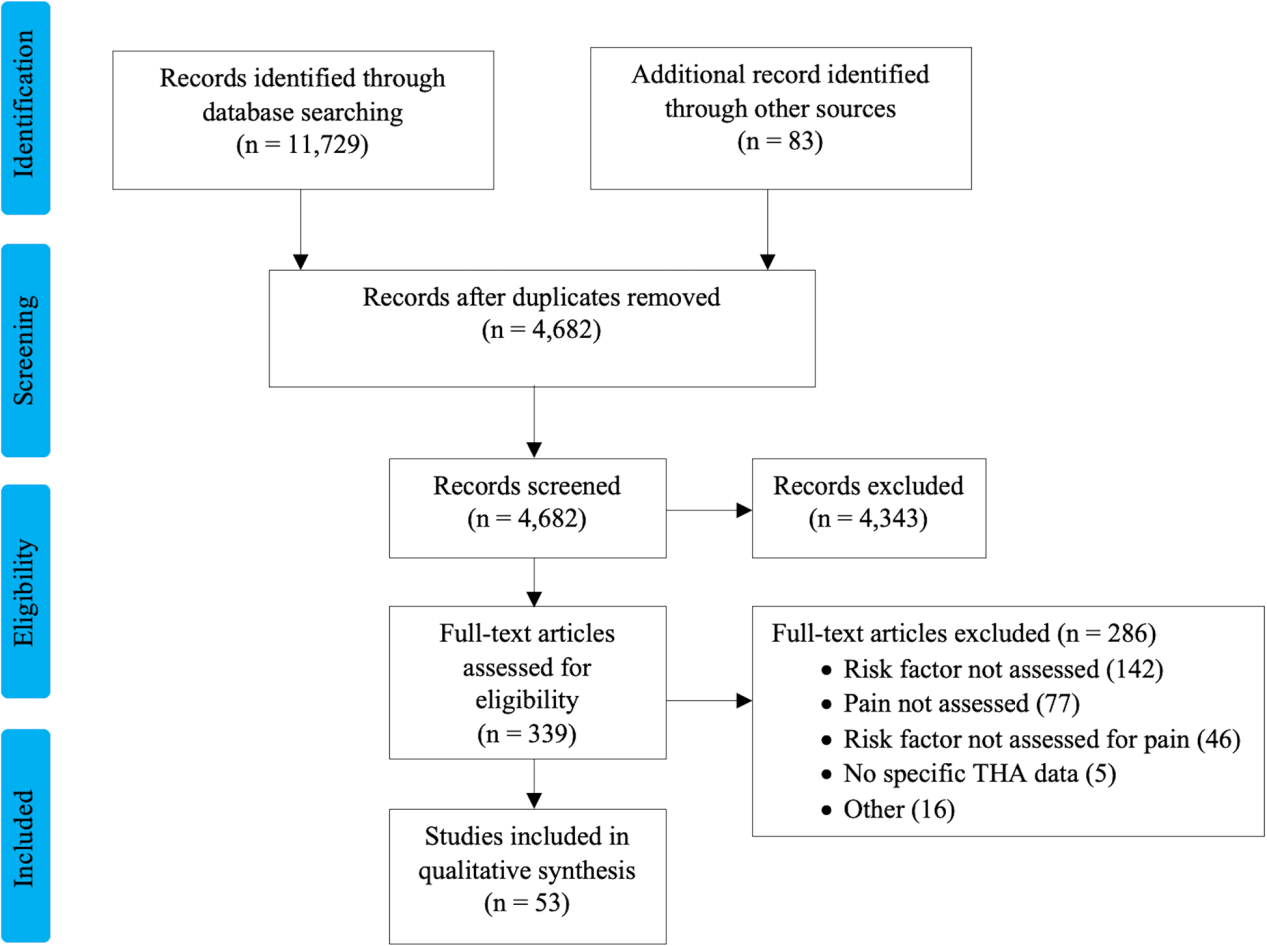
Flow diagram of a systematic review of articles published from January 1995 to May 2018 about adults undergoing total hip arthroplasty. The databases searched were PubMed (Medline), Scopus, Web of Science, Cumulative Index of Nursing and Allied Health Literature Plus (EBSCO), Embase, and Cochrane

A total of 11,729 studies were identified, including duplicates. The full-text review was performed for 339 publications, and 54 studies satisfied our inclusion criteria (Fig. 1). Most studies were observational cohort studies. The sample size ranged from 54 to 37,393 patients, and follow-up ranged from 24 h to 12 years. Other than in the studies by Busato et al. [7] and Röder et al. [8], THAs were performed in 1993 or later. Commonly evaluated risk factors for postoperative pain were preoperative pain and function (20 studies), medical and psychological comorbidities (18 studies), BMI (13 studies), socioeconomic status and ethnicity (12 studies), sex (11 studies), and radiographic severity of osteoarthritis (4 studies). Functional outcomes were assessed using the Western Ontario and McMaster Universities Arthritis Index (WOMAC), Oxford Hip Score (OHS), Harris Hip Score (HHS), and Hip Disability and Osteoarthritis Outcome Score (HOOS).

According to the bias criteria, 10 studies had a high risk of bias, 31 had a medium risk, and 13 had a low risk (Table 1). Most studies adjusted for confounders. However, because of the retrospective design of many studies, selection methods and follow-up rates often could not be assessed.

**Table 1:** Risk of bias among 53 studies of adult THA

**First author**	**Year**	**Consecutive patient selection**	**Multicenter enrollment**	**Adequate follow-up**^a^	**Adjustment for confounders**	**Risk of bias**
Prentice [9]	2019	Yes	Yes	Yes	Yes	Low
Goodman [10]	2018	Yes	No	Yes	Yes	Low
Bedard [11]	2017	-	Yes	-	No	High
Brembo [12]	2017	Yes	Yes	Yes	Yes	Low
Li [13]	2017	-	Yes	-	Yes	Medium
Pinto [14]	2017	Yes	-	-	Yes	Medium
Dowsey [15]	2016	Yes	No	Yes	Yes	Low
Goesling [16]	2016	-	No	Yes	Yes	Medium
Greene [17]	2016	-	Yes	-	Yes	Medium
Tilbury [18]	2016	Yes	No	No	Yes	Medium
Lavernia [19]	2015	-	No	No	Yes	High
Mannion [20]	2015	-	-	Yes	Yes	Medium
Nam [3]	2015	No	Yes	No	No	High
Rajamaki [21]	2015	No	No	Yes	Yes	Medium
Dowsey [22]	2014	-	No	-	Yes	High
Greene [23]	2014	-	Yes	No	Yes	Medium
Judge [24]	2014	-	Yes	-	Yes	Medium
Lavernia [25]	2014	Yes	No	-	Yes	Medium
Motaghedi [26]	2014	No	No	Yes	No	High
Petrovic [27]	2014		-	Yes	Yes	Medium
Singh [28]	2014	Yes	No	-	Yes	Medium
Haverkamp [29]	2013	Yes	-	Yes	No	Medium
Judge [2]	2013	Yes	Yes	Yes	No	Low
Krupic [30]	2013	-	Yes	-	Yes	Medium
Neuburger [31]	2013	-	Yes	-	Yes	Medium
Pinto [32]	2013	Yes	No	No	Yes	Medium
Singh [33]	2013	Yes	No	-	Yes	Medium
Singh [34]	2013	Yes	No	-	Yes	Medium
Jones [35]	2012	Yes	Yes	Yes	Yes	Low
Liu [36]	2012	No	Yes	-	Yes	Medium
Smith [37]	2012	Yes	-	No	Yes	Medium
Allen Butler [38]	2011	Yes	-	Yes	No	Medium
Clement [39]	2011	Yes	No	-	No	High
Clement [40]	2011	-	No	-	Yes	High
Lavernia [41]	2011	-	No	Yes	Yes	Medium
Lavernia [42]	2011	-	Yes	Yes	Yes	Low
Johansson [43]	2010	-	-	-	No	High
Riediger [44]	2010	Yes	-	No	No	High
Schafer [45]	2010	Yes	No	No	Yes	Medium
Singh [46]	2010	Yes	No	-	Yes	Medium
Lavernia [47]	2009	-	-	-	Yes	High
Quintana [48]	2009	-	Yes	Yes	Yes	Low
Rolfson [49]	2009	-	Yes	-	Yes	Medium
Busato [7]	2008	-	Yes	-	Yes	Medium
Kessler [50]	2007	Yes	-	Yes	Yes	Low
Röder [8]	2007	-	Yes	-	Yes	Medium
Moran [51]	2005	Yes	No	Yes	Yes	Low
Fortin [52]	2002	Yes	Yes	Yes	Yes	Low
Holtzman [53]	2002	-	Yes	No	Yes	Medium
Nilsdotter [54]	2002	Yes	No	Yes	No	Medium
Jones [55]	2001	Yes	Yes	Yes	Yes	Low
Nilsdotter [56]	2001	-	No	Yes	Yes	Medium
Meding [57]	2000	Yes	No	Yes	Yes	Low
Fortin [58]	1999	-	Yes	No	Yes	Medium

“-”: unable to assess

^a^Adequate follow-up was defined as a loss to follow-up of < 20% before 1 year or < 30% after 1 year

### Preoperative BMI

We found that high BMI was associated with worse pain outcomes or increased opioid use after THA in 9 of 13 studies, although conclusions varied regarding its clinical importance (Table 2). Of the studies, 5 had a low risk of bias, 7 had a medium risk, and 1 had a high risk.

**Table 2:** Associations of BMI with pain/opioid use after THA

First author	Year	Study design	*n*	Follow-up	BMI groups (% of patient)	Outcome measures	Associations of high BMI with postoperative pain/opioid use
Prentice [9]	2019	RC	12,560	1 yr	< 30 (60.8); 30–35 (24.5); > 35 (14.6)	Opioid usage	Greater postoperative opioid use after initial 90-day recovery period
Li [13]	2017	RC	2040	6 mo	25–29.9 (37); 30–34.9 (22); 35–39.9 (10); ≥ 40 (4)	PCS, HOOS	Worse pain at baseline and 6 months, more improvement postoperatively
Rajamaki [21]	2015	CC	54	1–2 yr	< 30 (53); 30–35 (31); > 35 (16)	Questionnaire	Higher proportion of patients with pain
Judge [24]	2014	RS	1431	1 yr	< 25 (33); 25–30 (42); 30–35 (18); 35–40 (5.5); > 40 (1.7)	OHS	Worse pain, low clinical difference
Motaghedi [26]	2014	PC	60	1 d	< 25 (33); 25–30 (33); > 30 (33)	VRS	No association
Judge [2]	2013	PC	1431	1–5 yr	< 25 (33); 25–30 (42); 30–35 (18); 35–40 (5.5); > 40 (1.7)	OHS	Worse pain
Jones [35]	2012	PC	231	3 yr	> 35 (14)	WOMAC	Worse pain at 6 months; no association at 3 years
Liu [36]	2012	CC	428	1 d	30^a^	VAS for pain	Worse pain, low clinical significance
Smith [37]	2012	PC	1318	3 yr	NA	HHS	Worse pain
Singh [46]	2010	CC	5707, 3289	2 yr, 5 yr	< 25 (24); 25–29.9 (39); 30–34.9 (24); 35–39.9 (8); ≥ 40 (1)	Questionnaire	Worse pain
Busato [7]	2008	RC	20,553	3, 6, 9, 12 yr	< 25 (38); 25–30 (44); > 30 (18)	Questionnaire	No association
Kessler [50]	2007	PC	67	10 d, 3 mo	< 25 (16); 25–30 (54); > 30 (30)	WOMAC	No association
Moran [51]	2005	PC	800	6, 18 mo	28^a^ (range, 14–49)	HHS	Worse pain

*BMI* Body mass index, *CC* Case control, *CI* Confidence interval, *HHS* Harris Hip Score, *HOOS* Hip Disability and Osteoarthritis Outcome Score, *NA* Not available, *OHS* Oxford Hip Score, *OR* Odds ratio, *PC* Prospective cohort, *PCS* Pain catastrophizing scale, *RC* Retrospective cohort, *SD* Standard deviation, *VAS* Visual analog scale, *VRS* Verbal rating scale

^a^Expressed as mean BMI

### Preoperative pain and hip function

We found that preoperative pain and function were significant predictors of pain or persistent opioid use after THA in 16 of 20 studies (Table 3). Of the studies, 2 had a low risk of bias, 14 had a medium risk, and 4 had a high risk.

**Table 3:** Associations of preoperative pain medication or opioid use and function with pain medication or opioid use after THA

First author	Year	Study design	*n*	Follow-up	Outcome measures	Associations of poor preoperative pain/function with postoperative pain/opioid use
Prentice [9]	2019	RC	12,560	1 yr	Opioid usage	Number of preoperative opioid prescriptions, preoperative NSAID use, back pain, and non-specific chronic pain associated with greater postoperative opioid use
Bedard [11]	2017	RC	37,393	1 yr	Opioid usage rate	Greater preoperative opioid use associated with greater postoperative opioid use
Pinto [14]	2017	PC	64	2 d	NRS	No association
Goesling [16]	2016	PC	331	6 mo	WOMAC	Greater preoperative opioid use associated with greater postoperative pain and opioid use
Dowsey [22]	2014	PC	835	1 yr	HHS	No association between preoperative pain and postoperative pain; conflicting results for association between preoperative function and postoperative pain
Petrovic [27]	2014	CC	90	1 d	NRS	Higher odds of postoperative pain
Singh [28]	2014	RC	3823	2, 5 yr	VAS for pain	Worse pain in patients using a walking aid
Haverkamp [29]	2013	PC	189	2.3 yr^a^	VAS, WOMAC	Worse pain
Judge [2]	2013	PC	1431	1–5 yr	OHS	Worse pain
Pinto [32]	2013	CC	48	4–6 mo	NRS	Higher odds of postoperative pain
Singh [33]	2013	RC	3823	2, 5 yr	HHS	Worse hip pain with preoperative ipsilateral knee pain
Liu [36]	2012	CC	428	1 d	VAS for pain	Higher odds of postoperative pain
Smith [37]	2012	PC	1318	3 yr	HHS	Worse pain
Johansson [43]	2010	PC	75	2 yr	WOMAC, HHS, SF-36	Worse pain
Lavernia [47]	2009	PC	127 ^†^	3 yr	WOMAC	Worse pain
Röder [8]	2007	RC	13,766	≤ 10 yr	VAS for pain	No association
Fortin [52]	2002	PC	86	2 yr	WOMAC	Worse pain
Holtzman [53]	2002	PC	1046	1 yr	VAS for pain	Higher odds of postoperative pain
Nilsdotter [56]	2001	PC	162	1 yr	WOMAC	Worse pain
Fortin [58]	1999	PC	116	6 mo	WOMAC	Worse pain

*CC* Case–control, *CI* Confidence interval, *HHS* Harris Hip Score, *NRS* Numerical rating scale, *OHS* Oxford Hip Score, *OR* Odds ratio, *PC* Prospective cohort, *PCS* Physical component summary, *RC* Retrospective cohort, *RR* Risk ratio, *SF-12* 12-Item Short-Form Health Survey, *SF-36* 36-Item Short-Form Health Survey, *VAS* Visual analog scale, *WOMAC* Western Ontario and McMaster Universities Arthritis Index

^a^Mean follow-up. ^†^Included THA and total knee arthroplasty cases

### Age

We found an association between patient age and pain or opioid use after THA in 9 of 12 studies, but these results were controversial (Table 4). Of these studies, 3 showed older age to be predictive of persistent pain, whereas 5 found young age to be predictive. One study concluded that two opposite age ranges studied were associated with worse pain, and three studies found no association. Notably, 4 studies had a high risk of bias, 3 had a medium risk, and 5 had a low risk.

**Table 4:** Associations of patient age with pain/opioid use after THA

First author	Year	Study design	*n*	Age, yr	Follow-up	Outcome measures	Associations of older age with postoperative pain/opioid use
Prentice [9]	2019	RC	12,560	67 (59–75)^b^	1 yr	Opioid usage	Less risk of postoperative opioid use
Bedard [11]	2017	RC	37,393	< 50, 2.7%	1 yr	Opioid use	Less risk of postoperative opioid use
Brembo [12]	2017	PC	223	69^a^ (41–91)	3 mo	WOMAC	Worse pain
Nam [3]	2015	PC	196	50^a^, SD = 7.1	2.9 yr	Pain scale of 0–5	Less odds of having pain
Dowsey [22]	2014	PC	835	68^a^ SD = 9.9	12 mo	HHS	Better pain
Judge [2]	2013	RC	1431	70^a^	1–5 yr	OHS	Worse pain in patients aged < 60 or > 70
Liu [36]	2012	CC	428	67^a^ SD = 11	1 d	VAS for pain	Better pain
Smith [37]	2012	PC	1318	68.5^a^ SD = 9.9	3 yr	HHS	Worse pain
Clement [39]	2011	PC	171	> 80	1 yr	OHS	No association
			495	65–74			
Quintana [48]	2009	PC	291	> 70	2 yr	WOMAC	Worse pain
			299	≤ 70			
Nilsdotter [54]	2002	PC	124	71^a^	1 yr	WOMAC	No association
Jones [55]	2001	PC	197	55–79 (83%), ≥ 80 (17%)	6 mo	WOMAC, SF-36	No association

*CC* Case–control, *CI* Confidence interval, *HHS* Harris Hip Score, *OHS* Oxford Hip Score, *OR* Odds ratio, *PC* Prospective cohort, *RC* Retrospective cohort, *SD* Standard deviation, *SF-36* 36-Item Short-Form Health Survey, *WOMAC* Western Ontario and McMaster Universities Arthritis Index

^a^Data presented as mean

^b^Data presented as median (interquartile range)

### Sex

Female gender was a predictor of worse pain or opioid use after THA in 9 of 11 studies (Table 5). There were no studies with a high risk of bias, and three had low risk.

**Table 5:** Associations of female sex with pain/opioid use after THA

First author	Year	Study design	*n*	Female sex, %	Follow-up	Outcome measures	Associations of female sex with postoperative pain/opioid use
Prentice [9]	2019	RC	12,560	59	1 yr	Opioid usage	Greater opioid use
Brembo [12]	2017	PC	223	71	3 mo	WOMAC	No association
Pinto [14]	2017	PC	64	59	2 d	NRS	Worse pain
Mannion [20]	2015	RC	261	50	1 yr	OHS, WOMAC, SF-12	No association
Petrovic [27]	2014	CC	90	47	1 d	NRS	Higher odds of pain
Liu [36]	2012	CC	428	58	1 d	NRS	Worse pain
Smith [37]	2012	PC	1318	NA	3 yr	HHS	Worse pain
Lavernia [41]	2011	RC	658	59	2 yr	HHS, SF-36, WOMAC	Worse pain
Singh [46]	2010	PC	5707, 3289	51	2 yr, 5 yr	Pain medication use	Greater pain medication use
Rolfson [49]	2009	RC	6158	57	1 yr	VAS	Worse pain
Quintana [48]	2009	PC	590	49	2 yr	WOMAC, SF-36	Worse pain

*CC* Case–control, *CI* Confidence interval, *HHS* Harris Hip Score, *NA* Not available, *NRS* Numerical rating scale, *NSAID* Nonsteroidal anti-inflammatory drug, *OR* Odds ratio, *PC* Prospective cohort, *RC* Retrospective cohort, *SF-12* 12-Item Short-Form Health Survey, *SF-36* 36-Item Short-Form Health Survey, *VAS* Visual analog scale, *WOMAC* Western Ontario and McMaster Universities Arthritis Index

### Radiographic severity of arthritis

Of the studies investigated, four examined the association between preoperative radiographic severity of arthritis and postoperative pain. We noted that 3 of these studies reported better pain outcomes in patients with severe arthritis, while one study found no predictive value (Table 6). Regarding risk of bias, 2 studies had a low risk and 2 had a medium risk.

**Table 6:** Associations of preoperative radiographic severity of arthritis with pain after THA

First author	Year	Study design	*n*	Radiographic grade (% patients)	Follow-up	Outcome measures	Associations of higher-grade osteoarthritis with postoperative pain
Tilbury [18]	2016	PC	302	mK-L grade: 1 or 2 (26) mild, 3 or 4 (74)	1 yr	HOOS, OHS, SF-36	Greater pain improvement
Dowsey [15]	2016	CC	382	mK-L grade: 2 (1.4), 3a (6.8), 3b (33), 4a (25), 4b (35)	1 and 2 yr	HHS	Greater odds of pain improvement
Nilsdotter [56]	2001	PC	162	OARSI grade: 3 (70), 2 (29), 1 (1)	1 yr	WOMAC, SF-36	No association
Meding [57]	2000	PC	1163	-independent scale	32 (6–93) mo^a^	HHS	Less pain

*CC* Case–control, *HHS* Harris Hip Score, *HOOS* Hip Disability and Osteoarthritis Outcome Score, *mK-L* modified Kellgren and Lawrence grade, *OARSI* Osteoarthritis Research Society International, *OHS* Oxford Hip Score, *PC* Prospective cohort, *SF-36* 36-Item Short-Form Health Survey, *WOMAC* Western Ontario and McMaster Universities Arthritis Index

^a^Expressed as mean (range)

### Socioeconomic status and race/ethnicity

Associations of various socioeconomic parameters and race/ethnicity with pain or opioid use after THA were assessed in 12 studies (Table 7). Three studies had a high risk of bias, 6 had a medium risk, and 3 had a low risk. Of these studies, 4 found worse pain in African Americans at 2–3.5 years postoperatively [10, 19, 38, 42]. Reports on the educational level were mixed. Three studies reported that low socioeconomic status was a risk factor for poor pain outcomes after THA [31, 38, 40].

**Table 7:** Associations of race/ethnicity and SES with pain/opioid use after THA

First author	Year	Study design	*n*	Follow-up, yr	Outcome measures	Associations of race/ethnicity and SES with postoperative pain/opioid use
Prentice [9]	2019	RC	12,560	1	Opioid usage	Higher opioid use in African Americans, lower opioid use in Asian (compared to white)
Goodman [10]	2018	RC	4170	2	WOMAC	Worse pain in African Americans
Lavernia [19]	2015	RC	564	3.5 (1–9)^a^	VAS, WOMAC, SF-36	Worse pain in African Americans
Dowsey [22]	2014	PC	835	1	HHS	No association
Greene [23]	2014	RC	11,464	1	VAS	Worse pain in patients with low education
Neuburger [31]	2013	RC	59,680	0.5	OHS	Worse pain in low SES
Krupic [30]	2013	RC	1216	1	VAS	Worse pain in immigrants
Allen Butler [38]	2011	PR	119	2	VAS, HHS	Worse pain in African Americans, those with low education, and those with low income
Lavernia [42]	2011	RC	739	2	WOMAC, SF-36	Worse pain in minority patients, especially African Americans
Clement [40]	2011	PC	1359	1	OHS	Worse pain in more deprived patients
Schafer [45]	2010	CC	1113	0.5	WOMAC	Greater odds of poor pain outcome in patients who are single, living alone, on disability
Fortin [58]	1999	PC	116	0.5	WOMAC	No association with education level

*CC* Case–control, *CI* Confidence interval, *HHS* Harris Hip Score, *OHS* Oxford Hip Score, *OR* Odds ratio, *PC* Prospective cohort, *PR* Prospective randomized, *RC* Retrospective cohort, *SES* Socioeconomic status, *SF-36* 36-Item Short-Form Health Survey, *THA* Total hip arthroplasty, *VAS* Visual analog scale, *WOMAC* Western Ontario and McMaster Universities Arthritis Index

^a^Data presented as mean (range)

### Preoperative comorbidities

Seventeen of 18 studies found a negative association between medical or psychological comorbidities and pain after THA or postoperative opioid use (Table 8). Of these, 2 articles had a high risk of bias, 11 had a medium risk, and 5 had a low risk. Psychological comorbidities were another frequently studied risk factor.

**Table 8:** Associations of preoperative medical/psychological comorbidities with pain/opioid use after THA

First author	Year	Study design	*n*	Follow-up	Outcome measures	Associations of preoperative comorbidities with postoperative pain/opioid use
Prentice [9]	2019	RC	12,560	1 yr	Opioid usage	Higher postoperative opioid prescriptions with anxiety, chronic pulmonary disease, substance abuse, acquired immunodeficiency syndrome (AIDS), peripheral vascular disease, chronic blood loss anemia, congestive heart failure
Bedard [11]	2017	RC	37,393	1 yr	Opioid use	Higher risk postoperative opioid use with preoperative anxiety, depression, drug use, alcohol use, smoking
Brembo [12]	2017	PC	223	3 mo	WOMAC	Worse pain with increased medical comorbidities
Dowsey [15]	2016	PC	382	1 and 2 yr	HHS	Worse pain with poor mental function
Greene [17]	2016	RC	17,147	1 yr	VAS for pain	Worse pain if using antidepressants
Rajamaki [21]	2015	PC	54	1–2 yr	NRS	Greater odds of pain in diabetes
Lavernia [25]	2014	RC	60	11 (3–24) mo	HHS	Worse pain in vitamin D insufficiency
Petrovic [27]	2014	CC	90	1 d	NRS	Higher odds of pain in type D personality, anxiety, depression
Dowsey [22]	2014	PC	835	1 yr	HHS	Worse pain with increased medical comorbidities
Pinto [32]	2017	CC	48	4–6 mo	NRS	Worse pain with poor disease process perception and emotional representation
Judge [2]	2013	PC	1431	1–5 yr	OHS	Worse pain in medical comorbidities
Singh [34]	2013	PC	5707, 3289	2 yr, 5 yr	VAS for pain	No association
Jones [35]	2012	PC	231	3 yr	WOMAC	Worse pain with cardiac disease
Smith [37]	2012	PC	1318	3 yr	HHS	Worse pain with cardiac disease, hypertension, increase medical comorbidities, NSAID use
Allen Butler [38]	2011	PR	119	2 yr	VAS, HHS, SF-12	Worse pain with poor mental component score
Singh [46]	2010	PC	5707, 3289	2 yr, 5 yr	Pain medication use	Greater odds of pain, NSAID use, and opioid use in depression
Riediger [44]	2010	PC	79	8 wk	WOMAC, SF-36	Worse pain in depression and somatoform disorders
Rolfson [49]	2009	RC	6158	1 yr	VAS for pain	Worse pain in anxiety and depression

*ASA* American Society of Anesthesiologists, *CAD* Coronary artery disease, *CC* Case–control, *CCI* Charlson Comorbidity Index, *CI* Confidence interval, *HHS* Harris Hip Score, *HTN* Hypertension, *NRS* Numerical rating scale, *NSAID* Nonsteroidal anti-inflammatory drug, *OHS* Oxford Hip Score, *OR* Odds ratio, *PC* Prospective cohort, *PR* Prospective randomized, *RC* Retrospective cohort, *SF-12* 12-Item Short-Form Health Survey, *SF-36* 36-Item Short-Form Health Survey, *VAS* 10-cm visual analog scale, *WOMAC* Western Ontario and McMaster Universities Arthritis Index

## Discussion

The most consistent association were found between poor pain outcomes and the female sex, high preoperative pain or low function, and various medical or psychiatric comorbidities. Females not only had worse pain at both short- and long-term follow-ups but also had higher odds of severe acute postoperative pain and long-term opioid use. Although this should not affect the patient selection, an effort should be made to optimize multimodal pain management in women to achieve better short-term pain control, decrease chronic pain, and minimize opioid dependence [59]. Postoperatively, multidisciplinary pain therapy has been shown to provide substantial pain relief and may be a valuable referral [60].

Preoperative pain and loss of function are two primary criteria for performing THA; therefore, they cannot be treated like other modifiable factors. A difference should be noted for patients using chronic pain medication, who are at higher risk of postoperative opioid dependence and may benefit from referral to a pain specialist preoperatively for intervention and a weaning protocol at the cost of delaying surgery [11, 16].

Patients with existing comorbidities are more reluctant to undergo elective surgeries [61]. In THA, they also experience increased complications requiring revisions [62]. Notably, patients with diabetes had eight times higher odds of having persistent pain than those without. Other medical comorbidities had a similar effect. Increased odds of acute and chronic pain, as well as opioid dependence, were repeatedly observed in patients with anxiety or depression. Our data suggest that preoperative medical optimization may be beneficial to pain outcomes. Additionally, treating those with active psychiatric conditions may also improve perceived pain and satisfaction [63].

Less consistent association was seen between poor pain outcomes and high BMI, low radiographic grade, and low socioeconomic status. The difference in pain outcomes among BMI groups was often small when compared with the overall improvement. Although most data show worse postoperative pain scores in patients with high BMI, this may be due to greater preoperative pain rather than a less surgical benefit. Nevertheless, weight loss may be beneficial in decreasing baseline pain, ultimately improving postoperative pain [64].

Advanced preoperative radiographic severity of osteoarthritis was mostly found to be associated with worse pain outcomes. Although the data were not robust, they support the current guidelines of attempting non-operative modalities for those with low-grade radiographic arthritis [65].

With respect to race and socioeconomic factors, most studies found worse pain outcomes in African Americans, immigrants, patients with low educational levels, and patients with low socioeconomic status. Additionally, African Americans and patients of lower socioeconomic status had worse pain on presentation. A lack of access to resources, along with health care disparities affecting these populations, likely contribute to a delayed presentation with greater pain from advanced disease or improper non-operative management [66]. Although it is encouraging that these patients achieved similar improvement from surgery as their counterparts, they may still benefit from attention to pain management and preoperative education.

The relationship between age and pain outcomes is less clear. Studies showed that older age could be associated with better, worse, or no difference in postoperative pain. Possible explanations for worse outcomes in younger patients include an increased level of activity and expectations [67]. Conversely, confounding comorbidities and poor recovery may contribute to persistent pain in older patients. Notably, two studies reported worse outcomes in older patients at 3- and 6-month follow-up, whereas, most studies at later time points showed no effect or the opposite. This may be attributed to decreased healing and rehabilitation potential in older patients who are less healthy and less active. Although results conflict, data exist showing both ends of the age spectrum having worse pain outcomes. We may consider holding off surgery in young patients due to worse pain outcomes, in addition to other complications such as early implant failure [68]. Older patients, if they meet surgical criteria, should not delay the operation, or the recovery and rehabilitation potential is diminished.

This is the first systematic review, to our knowledge, that assessed common preoperative risk factors for pain after THA. A strength of this study is that a large number of studies were included, most of which had a low or medium risk of bias. However, there are several limitations to consider. The majority of outcome-based studies are observational cohorts with varying quality and risk of bias. In retrospective studies, appropriate patient selection and loss of follow-up are difficult to assess. Additionally, not all studies adjusted for confounding factors. Most studies reported follow-up of < 2 years, and only two studies continued beyond 5 years. The effects, if any, that these risk factors may have beyond this time frame are impossible to evaluate. Additionally, it is apparent that there is no consensus on an outcome measure for postoperative pain. The differences in the various questionnaires may also be a source of bias. Although some studies reported the effect of risk factors to be small relative to overall improvement from surgery, the heterogeneity of study design and outcome measures prohibited a meaningful meta-analysis to determine the magnitude of effect for each predictive factor. Future outcomes research will benefit from standardized design and outcome measures that allow for meta-analysis and the production of stronger evidence.

Persistent or severe postoperative pain is often difficult to explain and remains a major detriment to overall patient satisfaction and recovery after THA. Identification and management of preoperative risk factors is crucial. Although age, sex, and certain socioeconomic elements cannot be altered, they provide value in the discussion of surgical benefits and patient expectations. Additionally, recognizing patients at higher risk of worse pain outcomes allows the provider to appropriately manage their pain. Over-prescription of opioids is becoming a dangerous epidemic, and more THA patients are relying on them by the year [11]. Referring patients at high risk of postoperative pain to specialists will provide safer and more reliable pain regimens. Finally, these risk factors hold value in surgical decision-making. Modifiable characteristics such as obesity, mental health, and medical comorbidities present the opportunity to improve pain outcomes with preoperative optimization.

## Conclusion

We have identified preoperative risk factors that were consistently predictive of greater postoperative pain after THA, despite the varying quality of studies that prohibit reaching concrete conclusions. Modifiable factors should be optimized preoperatively, whereas non-modifiable factors may be valuable to patient education, shared decision-making, and individualized pain management.

## Data Availability

The datasets used and/or analysed during the current study are available from the corresponding author on reasonable request. Original data in the form of articles used in this review are available with public access.
